# Mitochondria-Associated ER Membranes – The Origin Site of Autophagy

**DOI:** 10.3389/fcell.2020.00595

**Published:** 2020-07-16

**Authors:** Ming Yang, Chenrui Li, Shikun Yang, Ying Xiao, Xiaofen Xiong, Wei Chen, Hao Zhao, Qin Zhang, Yachun Han, Lin Sun

**Affiliations:** ^1^Hunan Key Laboratory of Kidney Disease and Blood Purification, Department of Nephrology, The Second Xiangya Hospital of Central South University, Changsha, China; ^2^Department of Nephrology, The Third Xiangya Hospital of Central South University, Changsha, China

**Keywords:** autophagy, mitophagy, mitochondria-associated endoplasmic reticulum membranes (MAMs), mitochondria, endoplasmic reticulum

## Abstract

Autophagy is a process of intracellular self-recycling and degradation that plays an important role in maintaining cell homeostasis. However, the molecular mechanism of autophagy remains to be further studied. Mitochondria-associated endoplasmic reticulum membranes (MAMs) are the region of the ER that mediate communication between the ER and mitochondria. MAMs have been demonstrated to be involved in autophagy, Ca^2+^ transport and lipid metabolism. Here, we discuss the composition and function of MAMs, more specifically, to emphasize the role of MAMs in regulating autophagy. Finally, some key information that may be useful for future research is summarized.

## Introduction

Autophagy is an evolutionarily conserved cellular process by which damaged organelles and excess proteins are degraded, and then, the decomposition products are recycled back to the cytoplasm (Mizushima and Komatsu, 2011; Kim and Lee, 2014; Onorati et al., 2018; Yang and Klionsky, 2020). Autophagy is classified into many types, such as mitophagy, lipophagy, and ribophagy, according to the cellular components or organelles that are degraded (Condello et al., 2019). Basal autophagy occurs in cells under normal conditions, and the level of autophagy changes during stress such as starvation (Fernandez et al., 2020). The development of a variety of diseases, such as inflammation (Racanelli et al., 2018) and cancer (Zeng and Ju, 2018), is accompanied by abnormal autophagy. Autophagy may be a potential target for disease treatment in the future. However, the molecular mechanism of autophagy, especially of the initiation and expansion of autophagosomes, has not been fully elucidated. Increasing evidence has shown that mitochondria-associated endoplasmic reticulum membranes (MAMs) are indispensable in the autophagy process, and many proteins that are directly involved in autophagy are located in MAMs (Betz et al., 2013). However, the relationship between MAMs and autophagy is not well understood. Here, we discuss the current evidence supporting the important role of MAMs in autophagy.

## Overview of MAMs

Previous studies have indicated that cellular organelles, such as mitochondria and the endoplasmic reticulum (ER), play independent biological roles. However, increasing evidence has shown that organelles are not independent structures, and it has been found that there is a physical connection between the ER and mitochondria, which has been named the MAMs (van Vliet and Agostinis, 2018). The relationship between mitochondria and the ER was observed in rat liver cells by Bernhard et al. (1952) and Bernhard and Rouiller (1956) and further observed by Copeland and Dalton in their studies of the pseudobranch gland of a teleost (Copeland and Dalton, 1959). It was not until 1990 when, due to the development of biological technology, Vance isolated “fraction X” from rat livers and named it the “MAMs” (Vance, 1990). Electron microscopy has shown that the ER and mitochondria can interact at a distance of approximately 10–20 nm (Csordas et al., 2006).

As a bridge between the ER and mitochondria, the MAMs are the dynamic connection that is composed of the subdomain of the ER, the outer mitochondrial membrane (OMM) and a series of proteins. Recently, more than 1,000 proteins have been found in MAMs fragments by mass spectrometric analysis (Sala-Vila et al., 2016). In addition, Del et al. (2017) used immunoprecipitation combined with a proteomic approach and revealed that the proteins that interact with AβPP on MAMs have the following main functions: mitochondrial function and lipid metabolism. Using peroxidase-mediated proximity biotinylation, Hung et al. identified 634 and 137 proteins on the ER and mitochondria, respectively. Upon Intersecting these proteins, 68 proteins were found to be localized to the MAMs (Hung et al., 2017). In addition to the above, mass spectrometric analysis of MAMs proteins has been performed by several laboratories (Zahedi et al., 2006; Poston et al., 2013; Ma et al., 2017; Wang et al., 2018). Since the MAMs are the signal communication platform, it mainly relies on proteins to perform its various functions, and the proteins located in MAMs are grouped according to their primary functions, for instance, Ca^2+^ transport: inositol 1,4,5-triphosphate receptor (IP3R) and voltage-dependent anion channel (VDAC1) (Tubbs et al., 2014; D’Eletto et al., 2018); lipid metabolism: acyl coenzyme A-cholesterol acyltransferase (ACAT) (Rusinol et al., 1994), acyl CoA:diacylgycerol acyltransferase 2 (DGAT2) (Stone et al., 2009); autophagy: autophagy related 14 (ATG14), autophagy related 5 (ATG5) (Hamasaki et al., 2013); and insulin signaling: protein kinase B (PKB), mammalian target of rapamycin complex (mTORC) (Betz et al., 2013; Rieusset, 2017). These multifunctional protein groups also suggest that the MAM play an important role in maintaining intracellular homeostasis and biological functions.

## Molecular Composition of the MAMs

### MAMs Tethers in Yeast

MAMs are the region of the ER that mediate communication between the ER and mitochondria (Annunziata et al., 2018; Lee and Min, 2018). The integrity of the MAMs is the basis of its biological function. Some proteins found in MAMs are involved in different biochemical reactions in the cell, while others are involved in maintaining the structural stability of the MAMs, and their absence destroys the integrity of the MAMs. The ER-mitochondria encounter structure (ERMES) is the protein complex that connects the ER and mitochondria in yeast cells (Kundu and Pasrija, 2020). The ERMES contains four core proteins: maintenance of mitochondrial morphology 1 (Mmm1), which is an anchoring ER protein; mitochondrial distribution and morphology protein 12 (Mdm12), which is a cytoplasmic junction protein; and Mdm34 and Mdm10, which are two OMM proteins (Lang et al., 2015). The physical Mmm1-Mdm12-Mdm34/Mdm10 interaction mediates efficient lipid transport, especially the transport of phospholipids between the ER and mitochondria (Kawano et al., 2018); an abnormal ERMES leads to dysregulated lipid exchange between the ER and mitochondria, resulting in abnormal cell growth (Kornmann et al., 2009).

## MAMs Tethers in Mammalian Cells

### Protein Complex-Mediated Tethers

The ER-mitochondria connections in mammalians are more complicated than those in yeast. The most important group of proteins involved in ER-mitochondria coupling is IP3R/Grp75/VDAC. IP3R is one of the most important calcium channels that is located in the ER and controls the release of Ca^2+^, thus affecting cellular metabolism and autophagy (Kania et al., 2017; Valladares et al., 2018). VDAC is a Ca^2+^-related protein located in the OMM that mediates the uptake of Ca^2+^ by mitochondria (Lipper et al., 2019). Grp75, a member of the heat shock protein 70 family, binds to IP3R and VDAC, improving the stability of the interaction and thus increasing the efficiency of Ca^2+^ transfer (Xu et al., 2018). Moreover, the sigma-1 receptor (Sig-1R) is a chaperone that is also located on MAMs and affects the transport of calcium ions between the ER and mitochondria by IP3R to increase the production of ATP (Hayashi and Su, 2007; Tagashira et al., 2014). In fact, the IP3R-VDAC1 complex is the core structure for calcium ion transport in MAMs, and this protein complex is also a marker of MAMs. We can use an *in situ* proximity ligation assay (PLA) to detect the integrity of the IP3R-VDAC1 complex to quantify MAMs (Tubbs and Rieusset, 2016; Zhu et al., 2017; Yang et al., 2019). In addition, the split-GFP-based contact site sensor (SPLICS) probe from the Cali laboratory can be used to measure the coupling between the ER and mitochondria (Cieri et al., 2018). The PTPIP51-VAPB interaction is also among the recently discovered set of interactions involved in ER-mitochondria coupling. Vesicle-associated membrane protein-associated protein B (VAPB) is a protein that is anchored to the ER membrane by a C-terminal transmembrane domain, and it plays an important role in the unfolded protein response and vesicle trafficking (Lee and Min, 2018). Protein tyrosine phosphatase-interacting protein 51 (PTPIP51) is a protein that is located in the OMM. PTPIP51 is a microtubule-associated protein, and it performs different biological functions by forming multiple protein structure complexes (Brobeil et al., 2017). Surprisingly, recent studies have shown that PTPIP51 forms protein complexes with VAPB in the MAMs that mediate calcium ion transport and autophagy (De Vos et al., 2012; Gomez-Suaga et al., 2017). The PTPIP51-VAPB complex can be regulated by other proteins. α-Synuclein is the central protein in the progression of Parkinson’s disease, and mutant α-synuclein disrupts the VAPB-PTPIP51 complex, resulting in the uncoupling of ER-mitochondria contacts and leading to dysregulated Ca^2+^ transfer and decreased mitochondrial ATP production in the development of Parkinson’s disease (Paillusson et al., 2017). TAR DNA-binding domain protein 43 (TDP-43) is a highly conserved and widely expressed nuclear protein (Chang et al., 2016). The accumulation of TDP-43 is associated with the development of various neurodegenerative diseases. Stoica et al. (2014) verified that overexpression of TDP-43 activated glycogen synthase kinase-3β (GSK-3β) by inhibiting its phosphorylation at serine-9, and that activated GSK-3β reduced binding of VAPB to PTPIP51, which resulted in disordered Ca^2+^ homeostasis. Another combination of proteins involved in ER-mitochondria conjugation is BAP31 and TOM40. B cell receptor-associated protein 31 (BAP31) is a transmembrane protein that is located in the ER and plays an important role in apoptosis and the endoplasmic reticulum-associated degradation (ERAD) pathway (Niu et al., 2017). The outer mitochondrial membrane 40 (TOM40) is the translocation enzyme complex on the OMM that promotes the translocation of external proteins into mitochondria (Gonzalez et al., 2018). Recently, Namba (2019) demonstrated that the connections formed by BAP31 and TOM40 facilitate pre-NDUFS4 transfer from the cytoplasm to the mitochondria, thereby increasing the activity of mitochondrial complex 1 and oxygen consumption. The protein combinations between the ER and mitochondria mentioned above play central roles in maintaining the structural integrity of MAMs.

### Individual Protein-Mediated Tethers

**FIGURE 1:**
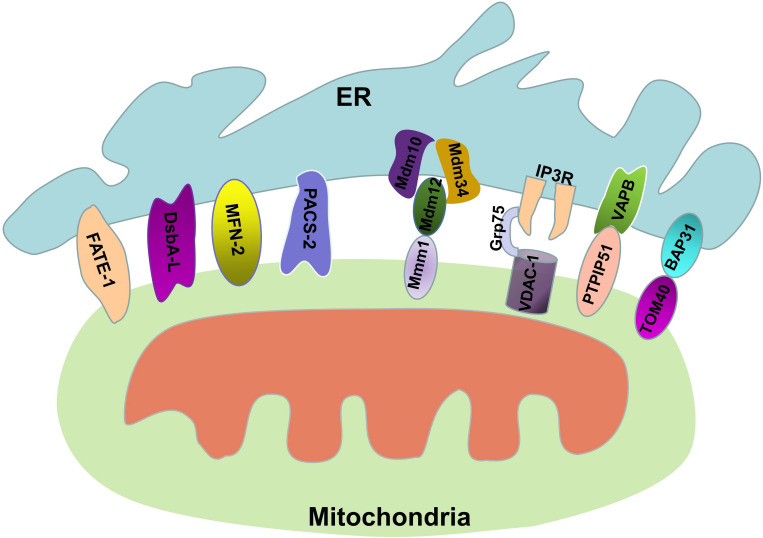
Protein connections involved in maintaining MAM stability. The MAM consists of parts of the OMM, ER subdomain, and some proteins. The structural stability of the MAM is maintained by these proteins.

In addition to the protein-protein interactions described above, some independent proteins in MAMs are also essential for maintaining ER-mitochondria conjugation. PDZD8 is an ER protein in metazoans that is functionally orthologous to Mmm1, and this protein is essential for maintaining the stability of the MAMs structure and contributes to calcium ion homeostasis in neurons (Hirabayashi et al., 2017). MFN2 is a GTPase that mediates mitochondrial fusion. In addition, MFN2 is also involved in respiration, autophagy and mitochondrial movement regulation, and in particular, MFN2 mediates the coupling of the mitochondria and the ER (Filadi et al., 2018). MFN2 in mitochondria is assembled into homo- or heterodimeric complexes with MFN2 in the ER when the mitochondrial fusion process is initiated (de Brito and Scorrano, 2008). When the expression of MFN2 is suppressed, the structure and function of the mitochondria are destroyed (Filadi et al., 2018). However, when MFN2 is overexpressed, the interaction of the ER and mitochondria is enhanced (Filadi et al., 2018), implying that MFN2 plays a significant role in the connection of the ER and mitochondria. Phosphofurin acidic cluster sorting 2 protein (PACS-2) is another protein that is involved in MAMs integrity, and it is also involved in apoptosis and autophagy (Herrera-Cruz and Simmen, 2017; Moulis et al., 2019). It has demonstrated that when PACS-2 is absent from cells, p20 is generated from BAP31 through a caspase-dependent pathway; p20 then induces mitochondrial fission by regulating Drp1, thus causing the destruction of MAMs integrity (Simmen et al., 2005). In contrast, overexpression of PACS-2 increases ER-mitochondria coupling (Herrera-Cruz and Simmen, 2017; Moulis et al., 2019). In fact, reducing the expression of PACS-2 will decrease the integrity of MAMs and inhibit the lipidation of LC3-II, thereby inhibiting autophagy (Hamasaki et al., 2013). Fetal and adult testis expressed 1 (FATE-1) is a testicular cancer antigen that is involved in uncoupling ER-mitochondria interactions and disrupting Ca^2+^ transfer from the ER to mitochondria, suggesting that it plays a role in regulating apoptosis (Doghman-Bouguerra et al., 2016). Because of the specific expression of FATE-1 in the testis and its low expression in other cells, the use of plasmids overexpressing FATE-1 is now a beneficial method for studying the effects of structural and functional changes of the MAMs on cell growth and metabolism. Parkin is also a protein that is involved in maintaining the integrity of MAMs; it is an E3 ubiquitin ligase and is associated with mitophagy (Wang et al., 2019). Ziviani (2018) demonstrated that Parkin alters MAMs integrity by affecting the ubiquitination of MFN2 (Basso et al., 2018). Recently, our group also verified that disulfide-bond A oxidoreductase-like protein (DsbA-L), which is a 25-kDa antioxidant enzyme that is also located in MAMs, inhibits the apoptosis of tubular cells in diabetic nephropathy by maintaining MAMs integrity (Yang et al., 2019; Figure 1). Under pathological conditions, the increase or decrease in ER-mitochondria connections caused by various factors leads to dysregulated intracellular communication signaling. However, the precise mechanism of MAMs regulation remains to be further studied.

## The Function of MAMs

Intracellular calcium homeostasis is the basis of cell metabolism. Concentrations of calcium ions in mitochondria that are too low can cause cellular energy metabolism disorders, while a high concentration of Ca^2+^ can cause cell death (Vakifahmetoglu-Norberg et al., 2017; Rossi et al., 2019). Normally, the ER releases Ca^2+^, which is then transported to the mitochondrial matrix, where it activates the tricarboxylic acid (TCA) cycle to stimulate ATP synthesis (Hurst et al., 2017). However, the transfer of excessive Ca^2+^ to the mitochondria leads to mitochondrial calcium overload, and to the opening of the mitochondrial permeability transition pore, leading to apoptosis (Pinton et al., 2008). Therefore, the MAMs act as the bridge between the ER and mitochondria, providing a buffer area for the transfer of calcium ions between the ER and mitochondria. It was Rizzuto et al. (1998) first uncovered the Ca^2+^ transfer function of the MAMs when they observed the spatial relation between the ER and mitochondria. When Ca^2+^ is released from the ER, the Ca^2+^ concentrations in some parts of the mitochondrial surface are much higher than those in most of the cytoplasm (Rizzuto et al., 1998); therefore, there is a structure between the ER and mitochondria that specifically transports calcium ions from the ER to mitochondria. To date, the importance of MAMs as calcium ion exchange platforms has been well established. After a cell is stimulated, the ER releases calcium ions through IP3R or ryanodine receptors (RyRs), which are the main Ca^2+^ channels of the ER (Wang et al., 2017; Laver, 2018). Because of the presence of MAMs, the ER and mitochondria have a spatial relationship with each other, thus allowing calcium ions to enter the mitochondria through VDAC1 on the OMM. Unlike the passive movement of Ca^2+^ through the OMM via the high-conductance protein VDAC1, Ca^2+^ movement through the inner mitochondrial membrane (IMM) is driven by an electrical gradient, and Ca^2+^ enters through the mitochondrial calcium uniporter (MCU). High Ca^2+^ concentrations on the mitochondrial membrane activate MCU to mediate the entry of Ca^2+^ into the mitochondrial matrix, which subsequently participates in a series of metabolic reactions (Nemani et al., 2018). As a regulatory protein, Grp75 maintains the stability of the interaction between IP3R and VDAC1, thus promoting the absorption of calcium ions by mitochondria (Xu et al., 2018).

In addition to the proteins mentioned above, there are many other proteins in the MAMs that are involved in Ca^2+^ transport. Sigma-1 receptor (Sig-1R) is a non-G-protein coupled chaperone of the ER that maintains the stability of IP3R to ensure appropriate Ca^2+^ signaling between the ER and mitochondria (Su et al., 2010). RNA-dependent protein kinase(PKR)-like ER kinase (PERK) is a key protein that is associated with ER stress, and is also abundant in MAMs. When this protein is knocked out, cells exhibit abnormal ER morphology, MAMs destruction, and calcium dysregulation (Verfaillie et al., 2012). Moreover, calnexin (CNX) is another MAMs protein that not only is involved in protein folding but also interacts with ER calcium pumps (Lynes et al., 2012). In summary, MAMs play an irreplaceable role in maintaining calcium homeostasis, and abnormal MAMs lead to dysregulation of intracellular signals and cause metabolic disorders.

In addition to Ca^2+^ transport, participation in lipid metabolism is also an important biological function of MAMs. Many of the enzymes involved in lipid metabolism, such as phosphatidylserine synthase (PSS) (Vance, 1990), ACAT (Rusinol et al., 1994), and DGAT2 (Stone et al., 2009), are located on MAMs. MAMs participate in lipid synthesis and transport by connecting the ER and mitochondria. However, this role of MAMs is not our focus here; please refer to Vance (2014).

## MAMs and Autophagy

Autophagy is a process of intracellular degradation in which redundant proteins or damaged components within the cell are isolated by a double-membrane vesicle called an autophagosome. Then, autophagosomes fuse with lysosomes to form autolysosomes, which are eventually degraded by lysosomal enzymes (Scrivo et al., 2018). The formation and development of autophagosomes involve a series of conserved genes called autophagy-related genes (ATGs), which encode proteins that regulate autophagy (Yu et al., 2018). The initiation of the isolation membrane is activated by the unc-51-like autophagy-activating kinase (ULK) complex, and then, the ULK complex activates the Vps34 complex by phosphorylating serine 14 of BECN1 (Ravikumar et al., 2010). This process occurs in the phosphatidylinositol 3-phosphate (PI3P)-rich subdomains of the ER. Subsequently, PI3P promotes the aggregation of various proteins (ATG18, ATG20, ATG21, and ATG24) in the assembly area, resulting in the growth of the phagophore. As the phagophore grows, some substances in the cell that need to be degraded are gradually enveloped by the phagophore (Sciarretta et al., 2018). Furthermore, the phagophore eventually matures and closes to form autophagosomes, and this process requires the participation of the ATG conjugation system. First, the ubiquitin-like protein ATG12 interacts with ATG5, and this interaction is mediated by ATG7, ATG10, and ATG3 (Sciarretta et al., 2018). The subsequent extension of the autophagosome is the result of the interaction of the ATG12-ATG5 complex with ATG16. This interaction promotes the lipidation of LC3 through its conjugation to phosphatidylethanolamine (Sciarretta et al., 2018), and LC3 promotes the maturation of the autophagosome. The mature autophagosome fuses with a lysosomes, and this fusion is mediated by soluble N-ethylmaleimide-sensitive fusion protein attachment protein receptor (SNARE) proteins, such as STX17, SNAP29, and VAMP8; this fusion forms the autolysosome (Nair et al., 2011; Diao et al., 2015).

## MAMs and the Initiation of Autophagy

The origin of autophagosomal membranes is still controversial, and the major source of membranes for autophagosome formation is unknown. Ge et al. (2017) demonstrated that the ER-Golgi intermediate compartment (ERGIC) is one of the membrane sources of autophagosomes. In addition, there is ample evidence that autophagy begins at the ER-mitochondria coupling site. As we described earlier, ERMES is the ER-mitochondria coupling complex in yeast that assembles via the Mmm1-Mdm12-Mdm34/Mdm10 interaction (Kawano et al., 2018). Surprisingly, ubiquitination of Mdm34 and Mdm12 is necessary for autophagy (Belgareh-Touze et al., 2017). This observation suggests that ER-mitochondria coupling is involved in the initiation of phagophore expansion. ATG14 is a component of the PI3K complex that is involved in autophagosome formation and is also a preautophagosome marker (Diao et al., 2015). Under starvation conditions, the content of ATG14 transferred to the MAMs increases, while ATG5, another marker of autophagosome formation, translocates to the MAMs until the autophagosome is formed, as observed by time lapse images in HeLa cells (Hamasaki et al., 2013). Further observation showed that when ER-mitochondria coupling is disrupted, ATG14 cannot be correctly localized in the MAMs, and the formation of autophagosomes is also inhibited (Hamasaki et al., 2013). This evidence fully demonstrated that MAMs play an irreplaceable role in the formation of autophagosomes, and the molecular mechanism underlying this phenomenon can also be explained by the functional roles of MAMs. Although existing studies have mainly focused on the effects of proteins on autophagosome formation, it is undeniable that lipids, especially phospholipids and sterols, also play an important role in the formation of autophagosomes. ATG8 (LC3), a ubiquitin-like protein, binds to the membrane and is a marker of isolation membrane expansion. Phosphatidylethanolamine (PE, the second most abundant phospholipid in mammalian cells) plays a very important role in this process. During isolation membrane expansion, ATG8 (LC3) is connected to PE by its C-terminal glycine residue. *In vivo*, PE is the main target of ATG8 (LC3), and a high level of PE can promote the connection between PE and ATG8, thereby facilitating the ATG8-mediated fusion and closure of the phagophore (Nair et al., 2012). Therefore, PE may be indispensable for the formation of autophagosomes. Moreover, another phospholipid, phosphatidylserine (PS, an important constituent of membrane structure in cells), can also act as a receptor of ATG8, and a small portion of ATG8 binds to PS (Sou et al., 2006). In addition, lipid droplets (LDs), which are the lipid storage organelles in cells, have been shown to be a critical source of lipids for the synthesis of autophagosomes (Li et al., 2015; Shpilka et al., 2015). In turn, the fatty acids (FAs) released in the process of autophagy are transferred into new LDs via DGAT1 to prevent FA-induced damage to the cell (Nguyen et al., 2017).

In addition to lipid synthesis, the dysregulation of Ca^2+^ in MAMs can also lead to abnormal autophagy (Ahumada-Castro et al., 2019). Elimination of etoposide-induced protein 2.4 (EI24), which is a protein located in the ER that is involved in regulating autophagy, destroys the integrity of the MAMs and inhibits autophagy in primary pancreatic β cells (Yuan et al., 2019). Further study has shown that when autophagy occurs, EI24 translocates to MAMs and interacts with the IP3R-Grp75-VDAC complex to maintain structural stability (Yuan et al., 2019). The disruption of Ca^2+^ signaling between the ER and mitochondria can interfere with the biological energy of cells and induce prosurvival autophagy (Cardenas et al., 2010). When ER-mitochondria Ca^2+^ transport is disrupted, AMPK translocates to MAMs and activates autophagy through BECN (Ahumada-Castro et al., 2019), which further confirms that the MAMs are the platform for the formation of autophagosomes. However, another group reached the opposite conclusion about the degree of MAMs integrity and autophagy. Disruption of the VAPB-PTPIP51 interaction through siRNA decreases the integrity of the MAMs, activates autophagy and overexpression of a synthetic protein that artificially increases ER-mitochondria coupling, which reduces the formation of autophagosomes (Gomez-Suaga et al., 2017). In terms of mechanism, VAPB-PTPIP51 coupling was found to affect autophagy by disrupting the transport of Ca^2+^ between the ER and mitochondria (Gomez-Suaga et al., 2017). Despite the seemingly opposite conclusion, we cannot deny the relationship between MAMs-mediated Ca^2+^ transport and autophagy.

ATG2 is also a key protein that regulates the expansion of phagophores; this protein has two subtypes, namely, ATG2A and ATG2B (Tang et al., 2017). During expansion, ATG2A translocates from the MAMs to the phagophore. ATG2A is anchored to the MAMs by a C-terminal, 45-amino-acid domain, which we called the MAMs localization domain (MLD), and TOM40 and TOM70 are responsible for the localization of ATG2A on the MAMs (Tang et al., 2019). Thus, a model was proposed in which the TOM40-TOM70 complex recruits ATG2 to the MAMs to transfer vesicular and/or non-vesicular lipids to the phagophore to enlarge the autophagosome and enhance autophagic flux (Tang et al., 2019). Similarly, promyelocytic leukemia protein (PML), which is a tumor suppressor, is located at the MAMs and controls the formation of autophagosomes by regulating the activity of the AMPK/mTOR/ULK1 pathway via affecting the transport of calcium ions from the ER to mitochondria (Missiroli et al., 2016).

## Pink/Parkin-Mediated Mitophagy

Currently, one of the best understood and most well-studied pathways of mitophagy is the PTEN-induced putative kinase 1 (PINK1) and Parkin(PARK2) pathway, which is associated with the development of Parkinson’s disease (Barodia et al., 2017; Truban et al., 2017). Under physiological conditions and through a mitochondrial targeting sequence, PINK is continuously transported to the mitochondria and degraded by matrix processing peptidases (MPPs). The degradation product is then cleaved by a protease located in the mitochondrial inner membrane, namely, presenilin-associated rhomboid-like (PARL), and cleaved PINK is transported back to the cytoplasm, where it is finally degraded in lysosomes (Ashrafi and Schwarz, 2013; Wang et al., 2020). In the pathological state, the cleavage of PINK is reduced because of mitochondrial damage. The non-cleaved PINK, via a process mediated by the OMM protein translocase of the outer membrane (TOM), accumulates on the outer membrane of the mitochondria. In addition, the level of mitochondrial pyruvate can also influence the aggregation of PINK on the OMM by promoting the direct interaction between PINK1 and TOM (Park et al., 2015). The accumulated PINK on the OMM phosphorylates serine 65 (Ser65) of ubiquitin, thereby recruiting Parkin. Subsequently, PINK phosphorylates and activates Parkin on the OMM, and then, activated Parkin polyubiquitinates proteins such as VDAC1 and p62/SQSTM1 (Wang et al., 2020). The ubiquitinated substrates bind to LC3 through LIR to recruit the autophagosomal membrane around the mitochondria, and then, further extension of the autophagosomal membrane leads to fusion with lysosomes to form mature mitochondrial autophagosomes and initiate the process of mitochondrial degradation (Tanida et al., 2008; Schaaf et al., 2016). Normal PINK/Parkin pathway-mediated mitophagy is the basis for intracellular homeostasis, and defects in this process are associated with many diseases, such as Parkinson’s disease (Nardin et al., 2016) and acute kidney injury (Lin et al., 2019). As the bridge between the ER and mitochondria, the most active organelle in the cell, what is the role of the MAMs in this process?

Yang and Yang (2013) demonstrated that ubiquitinated sites gradually undergo Parkin-mediated mitophagy, and the region between the ER and damaged mitochondria is where LC3 is recruited. Consistent with this observation, BECN1, the core component of the class III phosphatidylinositol 3-kinase (PtdIns3K) complex, is also found in MAMs, where it strengthens the connection between the ER and mitochondria and promotes the formation of autophagosome precursors (Gelmetti et al., 2017); therefore, MAMs are the site of the initiation of PINK/Parkin-dependent mitophagy. However, even though PINK, Parkin and BECN1 are found in MAMs, loss of PINK prevents BECN1 from accumulating in MAMs, and this process is independent of PARK (Gelmetti et al., 2017), which suggests a new role for PINK in regulating mitophagy. In addition, the overexpression of Parkin enhances the structure and function of the ER-mitochondria connection, promotes the transfer of Ca^2+^ from the ER to mitochondria and increases the production of ATP in mitochondria (Cali et al., 2013). Similarly, in Parkin mutant human fibroblasts, the integrity of the MAMs was destroyed, and further research has shown that PINK-mediated destruction of MAMs integrity was achieved by affecting the ubiquitination of MFN2 (Gautier et al., 2016; Basso et al., 2018). Glycoprotein 78 (gp78), a ubiquitin ligase (E3) anchored in the ER membrane that is associated with mitophagy (Guardia-Laguarta et al., 2019), has been confirmed to be located in MAMs (Wang et al., 2000). The available evidence suggests that the core protein involved in PINK/Parkin-mediated mitophagy is located in MAMs and is involved in the regulation of MAMs integrity and function.

## FUNDC1-Mediated Mitophagy

In mammalian cells, FUN14 domain containing 1 (FUNDC1) is involved in the receptor-mediated mitophagy pathway and is a highly conserved protein containing 155 amino acids (Zhang et al., 2017). FUNDC1 is located in the mitochondrial outer membrane protein-containing LC3-binding regions (LIR), and FUNDC1 recruits LC3 through LIR to initiate mitophagy during hypoxia (Liu et al., 2012). FUNDC1-dependent mitophagy is regulated by a variety of stress factors and cellular proteins. Under normoxic conditions, Tyr18 and Ser13 of FUNDC1 are phosphorylated by Src and casein kinase 2 (CK2), respectively, preventing it from binding to LC3 to induce autophagy (Liu et al., 2012; Chen et al., 2014). Under hypoxic conditions, the mitochondrial protein phosphatase PGAM5 mediates the dephosphorylation of Ser13, thus enabling FUNDC1 to bind to LC3 to form autophagosomes (Ma et al., 2020). In addition to Src and CK2, ULK1, which is a Ser/Thr kinase that participates in the formation of early autophagosomes, is also closely related to the mitophagy of FUNDC1. Under hypoxic conditions or treatment with FCCP, the expression of ULK1 increases, and ULK1 is recruited to the fragmented mitochondria; Moreover, transposable ULK1 interacts with FUNDC1 and promotes the phosphorylation of FUNDC1 at Ser17 to initiate autophagy (Wu et al., 2014). MARCH5 is a mitochondrial E3 ligase that can regulate mitophagy; Chen et al. demonstrated that MARCH5 directly interacts with FUNDC1, and degrades FUNDC1 by promoting its ubiquitination at lysine 119 and that the presence of MARCH5 induces the insensitivity of FUNDC1 to hypoxia-induced autophagy (Chen et al., 2017). FUNDC1 plays an essential role in receptor-mediated mitophagy, but what role does the MAMs play in this physiological process?

**FIGURE 2:**
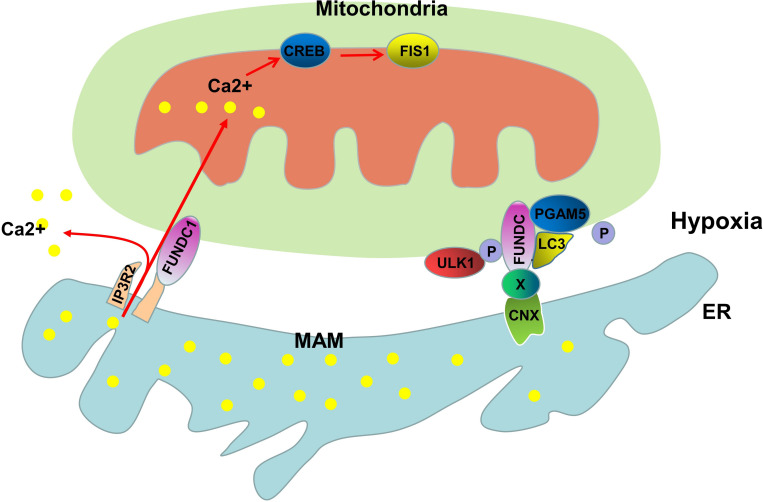
Molecular mechanisms of FUNDC1-mediated mitophagy. FUNDC1 is a MAM-associated protein that interacts with IP3R2 to regulate the expression of Fis1 through CREB. Under hypoxic conditions, FUNDC1 is transported to the MAM by CNX and unknown proteins (X). PGAM5-mediated dephosphorylation of Ser13 and ULK1-mediated phosphorylation of Ser17 promote the binding of FUNDC1 to LC3, leading to the initiation of mitophagy.

The direct relationship of the MAMs and FUNDC1-mediated mitophagy was demonstrated by Zhou et al., who showed that FUNDC1 is a MAM-localized protein that interacts with another MAMs protein, IP3R2, to mediate IP3R-dependent Ca^2+^ signaling from the ER to the mitochondria and cytosol (Wu et al., 2017). When the expression of FUNDC1 is decreased, the decreased intracellular Ca^2+^ levels inhibit the expression of Fis1 through Ca^2+^-sensitive cAMP-response element binding protein (CREB), thus causing mitochondrial dysfunction (Wu et al., 2017). Moreover, the decreased expression of FUNDC1 disrupts the interaction between the ER and mitochondria and reduces the protein abundance in MAMs (Wu et al., 2017). Another study of MAMs and FUNDC1 demonstrated that under normoxic conditions, there is a small amount of FUNDC1 in MAMs, and in response to hypoxia, FUNDC1 substantially accumulates in MAMs (Wu et al., 2016). What is the molecular mechanism by which FUNDC1 translocates to MAMs? CNX may play an indispensable role in this process. Immunoprecipitation experiments have shown that there is an interaction between the N terminus of CNX and the hydrophilic domain of FUNDC1. However, due to structural reasons, the N terminus of CNX is located in the lumen of the ER, and it is unlikely that the hydrophilic domain of FUNDC1 penetrates the lumen of the ER to interact with CNX; thus, there must be an unknown protein that mediates the interaction between CNX and FUNDC1. Depletion of CNX can inhibit FUNDC1 translocation to the MAMs under hypoxic conditions, which further confirms the role of CNX in FUNDC1 translocation (Wu et al., 2016; Figure 2). Although further studies on the role of MAMs in FUNDC1-mediated mitophagy are needed, the available evidence suggests that the MAMs provides a platform for FUNDC1 to perform its biological functions.

In addition to the proteins mentioned above, another protein involved in ER-mitochondria coupling, PACS2, also plays an important role in mitophagy. Coyne demonstrated that PACS2 mediates the integrity of the ER-mitochondria connection during stimulation with atherogenic lipids. The loss of PACS2 leads to MAMs destruction and dysregulated mitophagosome formation and mitophagy (Moulis et al., 2019).

## Conclusion

Autophagy is an important physiological process for the maintainance of cellular homeostasis, and defects in this process are associated with many diseases, such as Parkinson’s disease, cancer and acute kidney injury (AKI). As a bridge between the ER and mitochondria, the MAM also play an important role in Ca^2+^ transport, lipid metabolism and autophagy. On the one hand, the MAMs serve as the platform for autophagy-related proteins to perform their biological functions. On the other hand, the Ca^2+^ transport and lipid metabolism functions of MAMs may be involved in autophagosome development. Disruption of the structure and function of MAMs leads to abnormal autophagy. Although existing studies have fully demonstrated the correlation between the MAMs and autophagy, there are still many questions for us to explore. What proteins mediate the involvement of MAMs in the expansion of autophagosomes? What is the relationship between MAMs and diseases caused by abnormal autophagy? The available evidence supporting a relationship between MAMs and autophagy was obtained *in vitro*, and experiments are needed to further verify this phenomenon *in vivo*. It is believed that with the development of cellular and molecular biology technology, the regulatory mechanisms of MAMs and autophagy will be continuously elucidated, and the MAMs are expected to be an important target for the treatment of diseases related to autophagy.

## Author Contributions

MY, CL, YX, YH, XX, and WC designed and performed the study. HZ conceived the project. QZ and SY supervised the work. LS wrote the manuscript. All authors contributed to the article and approved the submitted version.

## Conflict of Interest

The authors declare that the research was conducted in the absence of any commercial or financial relationships that could be construed as a potential conflict of interest.
